# Emerging horizons on molecular and circulating biomarkers in pancreatic adenocarcinoma

**DOI:** 10.3389/fonc.2024.1483306

**Published:** 2024-11-07

**Authors:** Marta Moretti, Antonella Farina, Antonio Angeloni, Emanuela Anastasi

**Affiliations:** Department of Experimental Medicine, Sapienza University of Rome, Rome, Italy

**Keywords:** pancreas, adenocarcinoma, markers, diagnosis, target therapy

## Abstract

Pancreatic ductal adenocarcinoma (PDAC) is the most aggressive and invasive type of pancreatic cancer and is expected to soon become the second leading cause of cancer-associated death. The high mortality rate is due to the clinical features that allow asymptomatic progression to advanced stages, a period when current therapeutic treatments have limited efficacy. To address these challenges, researchers are focused on identifying new molecular and circulating markers for early PDAC detection and precision medicine. In this mini-review, we report the most well-known and recently identified molecular and circulating biomarkers. This study aimed to emphasize the need for continued innovative research to develop diagnostic algorithms and therapies to improve the management of patients with PDAC.

## 1 Introduction

Pancreatic ductal adenocarcinoma (PDAC) remains one of the most challenging and devastating cancers and is characterized by late detection and aggressive biology that often leads to therapeutic failure (1). The number of pancreatic cancer cases has been increasing steadily, making it the second leading cause of cancer-related deaths, surpassing breast cancer and projected to soon surpass colorectal cancer (2, 3). The poor prognosis of PDAC is primarily attributed to the lack of effective early detection methods and inherent chemoresistance (1, 4–6). Due to the broad genetic heterogeneity and dense stromal environment of pancreatic tumors, current treatment options, including surgery, radiation, chemotherapy, immunotherapy, and targeted therapies, have yielded only marginal improvements in overall survival (4). To address these challenges, recent PDAC research has focused on a wide range of biomarkers, exploring their potential from multiple perspectives for early detection and improved disease management (7, 8). Some studies have specifically examined circulating tumor DNA (ctDNA) levels in plasma samples from patients with early-stage (I-III) pancreatic adenocarcinoma who are potential candidates for surgery. This review, however, centers on biomarkers for early disease detection, aiming to enhance strategies for the timely diagnosis of PDAC. We explore these emerging biomarkers and highlight their potential to transform PDAC diagnosis and improve patient outcomes through earlier therapeutic interventions.

## 2 Molecular markers

### 2.1 Key molecular biomarkers

Molecular markers play a crucial role in the diagnosis and prognosis of several cancers. Among these, key markers such as KRAS, TP53, SMAD4 and CDKN2A have been identified in PDAC (10). The complex genetic and molecular landscape of PDAC, including its heterogeneity and tumor microenvironment, further highlights the importance of these markers (11).

#### 2.1.1 KRAS

Kirsten rat sarcoma viral oncogene homolog (KRAS) is the most well-known oncogene and has the highest mutation rate across all cancers (12). In PDAC, KRAS mutations serve as a major driver of the disease. These mutations are critical for PDAC development and progression, underscoring the importance of KRAS as a target for research and therapeutic interventions. In PDAC the most common alterations are predominantly found in codon 12, specifically G12D, G12V, and G12R (13, 14) and are often associated with poorer therapy response and overall survival (14). While G12D, G12V, and G12R mutations are the most frequent, G12R mutations have been linked to longer survival, particularly when not accompanied by PI3K pathway alterations (15). The development of small-molecule inhibitors targeting the KRAS pathway provides several treatment options for PDAC patients (16). Moreover, recent advancements have led to the development of irreversible covalent inhibitors for the KRAS-G12C variant, which have shown promise in clinical trials (17). Other strategies, such as targeting the MAPK signaling pathway to inhibit KRAS function, have also shown potential (18). Despite these advancements, challenges remain in developing small-molecule inhibitors that directly target KRAS, including the need for further optimization and the potential for resistance (19). However, the mutational status of KRAS may not be sufficient to identify tumors that are effectively dependent on it. Instead, the identification of specific KRAS-driven molecular biomarkers in certain PDAC subtypes may be more beneficial for tailoring targeted therapies (20). A subset of PDACs, known as KRAS-WT, exhibit a distinct molecular profile, including mutations in TP53 and BRAF, and are enriched with targetable alterations, suggesting the potential for expanded therapeutic options (21, 22).

#### 2.1.2 TP53

The TP53 gene, which encodes the tumor suppressor protein p53, has garnered significant attention as a molecular marker in PDAC. Notably, the inactivation of TP53, particularly in combination with KRAS activation, has been shown to drive the development of PDAC (23). TP53 mutations are a common in this neoplasm, with a prevalence of 50-75% (24, 25). These mutations, especially gain-of-function mutations, play a significant role in promoting disease progression, including metastasis (24, 26). The presence of TP53 mutations is also associated with a poorer prognosis and resistance to antineoplastic therapies (27). Therefore, detecting and characterizing TP53 mutations in PDAC could be crucial for predicting patient outcomes and guiding treatment decisions. Missense mutations in TP53 have been found to enhance fibrosis and create an immunosuppressive microenvironment in PDAC tumor, potentially contributing to treatment resistance (28). Mutant TP53 can also alter the tumor microenvironment and immune responses, further promoting PDAC progression (26). Additionally, the introduction of wild-type TP53 has been found to increase the sensitivity of PDAC cells to various treatments, suggesting a potential therapeutic strategy (26).

#### 2.1.3 SMAD4 (DPC4)

SMAD4, also known as DPC4 (Deleted in Pancreatic Cancer 4), belongs to a family of signal transduction proteins and it is considered a pivotal tumor suppressor gene involved in the TGF-β signaling pathway, which regulates cell growth, differentiation, and apoptosis. It is frequently inactivated in PDAC (29). Protein inactivation occurs either through deletion or mutation, making SMAD4 a critical molecular marker for this malignancy. SMAD4, has been suggested as a valuable diagnostic marker for PDAC, with higher expression compared to other cancers (30). Its loss is significantly associated with poor prognosis (31), and patients with SMAD4-expressing PDACs tend to have longer survival following surgical resection (32). The presence or absence of SMAD4 in PDAC significantly influences the response to specific therapies. SMAD4-positive tumors have been found to respond better to neoadjuvant therapy, with lower rates of lymph node metastasis (33, 34). SMAD4 status also affects the response to radiation therapy, with SMAD4-deleted PDAC showing worsened disease-free survival (35). Furthermore, SMAD4 loss has been associated with a response to neoadjuvant chemotherapy with the autophagy inhibitor hydroxychloroquine (36). These findings highlight the potential of SMAD4 as a marker for predicting treatment response and guiding personalized therapeutic strategies in PDAC.

#### 2.1.4 CDKN2A (p16)

Another common genetic alteration in PDAC is the deletion or mutation of the CDKN2A gene, which encodes the p16 tumor suppressor protein. Studies have shown a higher frequency of deletions and mutations in this gene in PDAC cell lines compared to primary tumors (37, 38). These alterations, including frameshift, nonsense, and missense mutations, as well as homozygous deletions, are associated with the development and progression of the disease (6, 39). The presence of these alterations can also impact the biological behavior and clinical staging of PDAC, potentially aiding in prognosis (39). These findings underscore the importance of genetic and epigenetic alterations, including those in the CDKN2A gene, in the development and progression of this malignancy (40).

### 2.2 New potential biomarkers

A promising avenue in cancer research focuses on identifying specific molecular alterations unique to pancreatic ductal adenocarcinoma (PDAC). These include genetic mutations, epigenetic changes, and abnormal signaling pathways that may serve as targets for early detection and individualized treatment. Recent investigations have highlighted potential molecular biomarkers for PDAC, with the most promising candidates being BRCA1/2, MLL, as well as Gastrokine proteins and Nucleoporins. These emerging markers offer new opportunities for developing targeted therapies and improving diagnostic precision in PDAC management. Recent studies have identified promising therapeutic targets and diagnostic biomarkers in PDAC, including CCNB1, FHL, HLA-DPA1, and TUBB1. These markers may shed light on the underlying molecular mechanisms of pancreatic cancer and contribute to the development of more precise diagnostic tools and targeted treatments. Their role in cell cycle regulation, immune response, and tumor progression underscores their potential in enhancing early detection and therapeutic strategies, offering new hope for improving patient outcomes in this aggressive malignancy (41). However, further in-depth research is essential to validate the clinical utility of these markers and ensure their successful integration into standard diagnostic and therapeutic regimens. This step is critical to confirming their efficacy, reliability, and potential in improving patient outcomes, particularly in early detection and treatment personalization for PDAC.

#### 2.2.1 BRCA1/2

The tumor suppressor genes BRCA1 and BRCA2 are involved in essential cellular functions necessary for cell replication and DNA synthesis, playing a crucial role in DNA repair and tumor suppression (31). BRCA1/2 mutations, present in approximately 10% of pancreatic cancer cases, have emerged as important biomarkers (42). Mutation in BRCA1 and BRCA2 are associated with an increased risk of pancreatic cancer (43–45). BRCA1 expression patterns in PDAC may serve as potential prognostic biomarkers, with cytosolic BRCA1 distribution linked to higher pathologic stage and potentially decreased recurrence-free survival (31). BRCA-associated pancreatic cancers have demonstrated greater sensitivity to platinum-based chemotherapy and PARP inhibitors, with patients showing partial responses to these treatments (45–47).

#### 2.2.2 MLL

The Mixed-Lineage Leukemia (MLL) family of proteins, which includes several histone methyltransferases such as MLL1 (KMT2A), MLL2 (KMT2B), MLL3 (KMT2C), and MLL4 (KMT2D), plays a crucial role in regulating gene expression through histone modifications, particularly the methylation of histone H3 lysine 4 (H3K4). These modifications are critical for controlling chromatin structure and gene transcription, which are essential for normal cellular function and development (48). The role of the MLL family in the context of PDAC is attracting considerable interest due to its involvement in epigenetic regulation and cancer progression. However, the use of MLL as a molecular marker in PDAC remains a topic of ongoing debate, with some studies suggesting that mutations in MLL1, MLL2 and MLL3, are associated with improved survival (10). The MLL1-H3K4me3 axis has been implicated in regulating PD-L1 expression and immune evasion in pancreatic cancer, suggesting a potential role for MLL in this context as well (49).

#### 2.2.3 Gastrokine proteins

Gastrokines (GKNs) are stomach-specific proteins with potential roles in gastric mucosal homeostasis and tumor suppression (50). The loss of GKN expression in various cancers, combined with its antiproliferative effects, suggests a tumor suppressor role for these proteins (50–52). GKN1 is abundantly expressed in normal gastric epithelium but is downregulated in gastric adenomas and carcinomas (52, 53). Additionally, overexpression of GKN1 in gastric cancer cells inhibits proliferation and induces cell death (52). Recent research has extended these findings to pancreatic cancer, showing that GKN proteins are expressed in early lesions of pancreatic intraepithelial neoplasm (PanIN), but are absent in healthy pancreas and invasive cancer (51). GKN1 and GKN2 appear to delay pancreatic carcinogenesis by promoting apoptosis and inhibiting proliferation in precursor lesions (51).

#### 2.2.4 Nucleoporins (Nup170 and Nup160)

Nucleoporins (Nups) are proteins considered the building blocks of the nuclear pore complexes and have been linked to a multitude of cancers through nucleo-cytoplasmic cargo trafficking, cell division, signaling pathways, chromatin-related processes, and protein stability and degradation (54). Regarding PDAC, Nup 170 and Nup160, have been identified as potential molecular markers for the early diagnosis this neoplasm (55). Furthermore, the genetic inactivation of Nupr1, a key nucleoporin that mediates stress response in the pancreas, and is frequently upregulated in pancreatic cancer, has been shown to suppress malignant transformation and influence the development of different PDAC subtypes (56). These findings suggest that nucleoporins play a significant role in PDAC progression and may have potential as therapeutic targets.

#### 2.2.5 CCNB1

The gene encoding the protein Cyclin B1 (CCNB1), an important cell cycle regulator involved in mitosis, has recently been identified as a potential prognostic biomarker in PDAC (57). Bioinformatics analyses of gene expression data revealed CCNB1 as a significantly upregulated gene in PDAC tissues (41, 57). CCNB1 was found to be involved in key cellular processes such as vesicle organization, lymphocyte activation, and adaptive immune responses (41). Validation studies confirmed the differential expression of CCNB1 in PDAC compared to normal tissues, supporting its potential as a diagnostic and prognostic marker (57). Additionally, the inhibition of Cyclin Dependent Kinase-1 (CDK-1), a protein that interacts with Cyclin B1, has shown promise as a novel therapeutic strategy against PDAC (58). These findings collectively underscore the potential of targeting CCNB1 and its associated proteins in the treatment of this neoplasm.

#### 2.2.6 FHL

The Four and a Half LIM (FHL) family is a group of proteins characterized by the presence of LIM domains, which are double zinc finger motifs involved in protein-protein interactions. These proteins play critical roles in various cellular processes, including gene expression, cell adhesion, cytoskeletal organization, and signal transduction. FHL plays a significant role in PDAC progression and treatment resistance. In particular, FHL1 can act as both a tumor suppressor and an oncogenic protein, depending on its phosphorylation status and interactions with various signaling pathways (59). In PDAC, FHL1 expression is regulated by Immortalization-upregulated protein (IMUP) (60) and Nucleophosmin1 (NPM1) (61): depletion of IMUP increases FHL1 expression, while overexpression of NPM1 reduces it through promoter methylation (62). FHL2, in PDAC, has been shown to play a crucial role in cell survival, proliferation, and radio-resistance (63). Indeed, overexpression of FHL2 is associated with tumor metastasis in PDAC (64). In contrast, its depletion leads to reduced cell survival and increased apoptosis (63). Finally, FHL3 has also been reported to promote PDAC invasion and metastasis by inhibiting ubiquitin degradation of EMT-associated transcription factors (65). The findings highlights that these proteins may serve as promising therapeutic targets in PDAC.

#### 2.2.7 HLA-DPA1

HLA-DPA1 belongs to the human leukocyte antigen (HLA) class II alpha chain paralogs and plays a central role in the immune system by presenting peptides derived from extracellular proteins. In PDAC, the expression of classical HLA class II antigens, including HLA-DPA1, has been associated with a poor prognosis (66). Similarly, high expression of HLA-G, a tolerogenic molecule implicated in tumor escape, has been linked to a poor prognosis in PDAC (66). These findings suggest a potential role for HLA-DPA1 in disease progression and the immune response, warranting further investigation.

#### 2.2.8 TUBB1

TUBB1 Albahde (2020) found that TUBB1, a member of the tubulin superfamily, is overexpressed in PDAC, with its downregulation leading to reduced cellular proliferation, invasiveness, and tumor growth (67). Recent bioinformatics analyses of gene expression data have revealed several hub genes, including TUBB1, as potential biomarkers in PDAC (41). These genes are involved in various cellular processes, such as vesicle organization, lymphocyte activation, and adaptive immune responses. Collectively, these findings suggest that TUBB1 may serve as a potential therapeutic target and prognostic marker in PDAC.

## 3 Circulating biomarkers

Over the past decade, a wide range of potential circulating biomarkers has been described for PDAC, with serum being the most utilized matrix due to its ease of collection and non-invasive nature. Despite their routine use, current biomarkers such as CA19-9 and CEA exhibit significant limitations in sensitivity and specificity. Consequently, their clinical application is confined to monitoring treatment response and recurrence in diagnosed patients, and they remain insufficient for screening or early diagnosis, which is a critical challenge. To streamline current research findings, we provide an updated review of established PDAC biomarkers.

### 3.1 Currently used biomarker

#### 3.1.2 CA19-9

Carbohydrate antigen 19-9 (CA19-9), also known as sialyl Lewis a (sLea), is currently the only widely used and approved biomarker for the pancreatic cancer diagnosis and monitoring (68, 69). However, CA19-9 has several limitations, such as yielding false negatives in patients with a Lewis blood type-negative phenotype and false positives in patients with obstructive jaundice (70). Moreover, CA19-9 is not tumor-type-specific, as its elevation can be observed in various malignancies, including those originating from the colorectum, gastric system, lung, breast, and liver, as well as in pancreatic neuroendocrine tumors (71). However, with the new frontiers of scientific research, despite its limitations, CA19-9 is finding new diagnostic roles, especially when used in combination with other biomarkers (71). In this regard, in PDAC, CA242 serum levels highly correlate with CA19-9 (72, 73), however, its complementary role to CA19-9 in the early diagnosis of PDAC remains unclear.

One of the most intriguing new functions of CA19-9 lies in its ability to accelerate pancreatic cancer progression by glycosylating proteins, binding to E-selectin, enhancing angiogenesis, and mediating the immunological response. This renders CA19-9 an attractive therapeutic target for cancer. Currently, therapeutic strategies employing CA19-9 to treat pancreatic cancer include using this molecule to develop specific anti-CA19-9 monoclonal antibodies that enable antibody-dependent cell-mediated cytotoxicity (ADCC) (71, 74). Additionally, CA19-9 can be targeted for drug delivery by binding it to nanoparticles (75). Another approach involves disrupting its biosynthesis by interfering with FUT3, a fucosyltransferase involved in the synthesis of Lewis antigens, and other enzymes (76). Therefore, targeting CA19-9 may offer novel therapeutic options, as interrupting CA19-9 could hinder the development and progression of PDAC.

#### 3.1.3 CEA

Carcinoembryonic antigen (CEA) is a glycoprotein typically produced by normal cells during embryonic development and tends to increase in presence of inflammatory processes or in tumors of the gastrointestinal tract. Due to its lower sensitivity and specificity compared to CA19-9 in early diagnosis of PDAC (77, 78), this biomarker cannot be relied upon for solitary diagnostic use. However, it may help to discriminate between benign and malignant pancreatic lesions when used in combination with CA19-9 (79), particularly in cases of advanced PDAC (80).

### 3.2 New potential circulating biomarkers

Due to the aggressive nature and rapid progression of PDAC, early diagnosis presents a significant challenge. The swift development of the tumor underscores the urgent need for effective biomarkers that can facilitate timely detection and improve patient outcomes. As a result, research efforts are increasingly focused on identifying novel biomarkers for early detection. Several molecules, either alone or in combination with conventional markers, have shown significant potential in improving the early diagnosis of this neoplasm. Among the circulating molecules currently under investigation, matrix metalloproteinases (MMPs) and their tissue inhibitors (TIMP-1), growth differentiation factor 15 (GDF15), and protein induced by vitamin K absence (PIVKA II) have garnered particular attention for their potential in enhancing diagnostic sensitivity and specificity compared to traditional markers. These promising biomarkers could play a crucial role in early diagnosis and personalized therapy of PDAC.

#### 3.2.1 MMPs

Matrix metalloproteinases (MMPs) are a family of zinc-dependent endopeptidases crucial for the remodeling of the extracellular matrix (ECM) (81). Initially thought to promote pre-metastatic development by degrading the ECM and basement membranes, MMPs also release soluble factors for cellular recruitment and express growth factor receptors in metastatic cells. Recent studies reveal that MMPs play a role in all stages of tumor progression, influencing signaling pathways, cytokine regulation, tumor growth, neo-angiogenesis, and cancer spread (82). During PC progression, MMPs drive both tumor growth and metastasis, making changes in their expression important surrogate markers. Pharmacological treatment or genetic ablation has been shown to reduce MMP levels, suggesting their potential anti-tumorigenic effects, which are currently under study (83). Regarding their role as biomarkers in Pancreatic cancer, combining MMP-7 with CA19-9 in periampullary carcinoma patients achieved a 100% positive predictive value (84). Additionally, Kahlert et al. found that serum levels of MMP-7 and MMP-12 are strong classifiers for diagnosing Pancreatic cancer compared to healthy controls (85).

#### 3.2.2 TIMP-1

Tissue inhibitors of metalloproteinases (TIMPs) play a crucial role in regulating extracellular matrix (ECM) turnover, tissue remodeling, and cellular behavior through interactions with MMPs. Maintaining this balance is critical, as disruptions can signal cancer progression (86). Among the TIMPs, TIMP-1 is notably linked to cancer, influencing cell proliferation, apoptosis, and differentiation. TIMP-1 also interacts with activated neutrophils via CD63, activating the ERK signaling pathway and promoting the formation of neutrophil extracellular traps (NETs) (87). In PDAC, TIMP-1 is linked to gemcitabine (GEM) resistance, although the impact of TIMP-1 downregulation in combination with GEM treatment remains unclear (88).

One of the most intriguing aspects of TIMP-1 lies in its ability to interact with activated neutrophils by binding to its receptor CD63. This interaction activates ERK, and the noncanonical TIMP1/CD63/ERK signaling axis induces the formation of NETs (87). This process accelerates tumorigenesis, progression, and therapy resistance in PDAC, where NETs frequently co-localize with high TIMP-1 expression (87, 89). In PDAC patients, TIMP-1 levels correlate with NET markers such as DNA-bound myeloperoxidase, suggesting an enhancement of prognostic accuracy when combined with CA19-9 (87). Additionally, TIMP-1 plasma levels are associated with the monocyte activation marker CD163, indicating that these proteins, when combined, could serve as powerful prognostic indicators (90).

#### 3.2.3 GDF15

Growth differentiation factor-15 (GDF-15), also known as MIC-1, belongs to the TGF-β superfamily and is typically undetectable under normal physiological conditions but its expression increases during cellular stress and pathological states (91). Originally identified in macrophages, elevated GDF-15 levels have been linked to various malignancies, making it a potential biomarker for disease (92–94). In response to stress signals, GDF-15 is released as a dimer through a specific secretory pathway, binds to the extracellular matrix, and undergoes cleavage into its active forms (95). It binds with high affinity to the GDNF family receptor α-like (GFRAL), which forms a complex with the tyrosine kinase co-receptor RET, activating signaling cascades involving the AKT, ERK1/2, and PLC γ pathways, but not the SMAD pathway (96). Elevated GDF-15 levels also influence immunological checkpoints, such as PD-1/PD-L1 (91), contributing to tumor development and suggesting its potential as a diagnostic marker for PDAC (96).

GDF-15 has also been implicated in the development of chemoresistance in PDAC. High levels of GDF-15 have been associated with resistance to commonly used chemotherapy drugs for PDAC, such as gemcitabine (GEM). This resistance mechanism can hinder the effectiveness of chemotherapy and worsen patient outcomes. Targeting GDF-15 signaling pathways has emerged as a potential therapeutic strategy for PDAC. Inhibition of GDF-15 or its downstream effectors may help inhibit tumor growth, metastasis, and chemoresistance in PDAC. Research efforts are underway to develop GDF-15-targeted therapies for the treatment of PDAC. Further research into the molecular mechanisms underlying GDF-15’s role in PDAC is needed to develop effective targeted therapies and biomarker-based diagnostic approaches (97).

#### 3.2.4 PIVKA II

Protein Induced by Vitamin K absence or Antagonist-II (PIVKA-II), also known as DCP (Des-γcarboxy prothrombin), is an abnormal form of prothrombin, the precursor of Coagulation Factor II, and its synthesis is Vitamin K-dependent. Ten glutamic acid residues in prothrombin undergo carboxylation by reduced Vitamin K; in its absence, PIVKA-II accumulates instead of maturing into prothrombin, thus indicating Vitamin K deficiency (98, 99). Initially identified with elevated levels in hepatocellular carcinoma (HCC), PIVKA-II has also been found in various gastrointestinal neoplasms, such as PDAC, colorectal cancer (CRC), and cholangiocarcinoma (98, 100). However, the mechanisms underlying PIVKA-II overexpression in these cancers, including HCC and PDAC, remain incompletely understood. While genetic mutations are not the primary cause, recent evidence points to factors such as hypoxia in the microenvironment, reduced gamma-glutamyl carboxylase activity, and altered Vitamin K metabolism contributing to PIVKA-II hyperproduction (101–104). Additional factors implicated include the downregulation of the VKORC1 gene in HCC and increased PARP-1 activity stimulating prothrombin gene transcription, potentially explaining elevated PIVKA-II in this cancer (105, 106). Although research has predominantly focused on HCC, similar mechanisms likely underlie PIVKA-II elevation in PDAC and other gastrointestinal cancers. Recent studies indicate PIVKA-II’s potential as a diagnostic biomarker in PDAC, showing promising diagnostic performance compared to CA19-9 and CEA (107). PIVKA-II not only helps distinguish benign and malignant pancreatic conditions but also aids in monitoring disease recurrence post-surgery, with levels decreasing following resection (108). Studies also suggest PIVKA-II may serve as a predictive factor for vascular invasion in PDAC (99). While its exact role in PDAC is still being elucidated, *in vitro* studies using PANC-1 cells suggest PIVKA-II secretion correlates with glucose-induced epithelial-mesenchymal transition (EMT), hinting at its role in disease aggressiveness (109). These observations have also been reinforced by a recent study that highlighted the association of elevated PIVKA-II levels with increased vimentin levels in the serum of PDAC patients (110). Taken together, these results open new frontiers in the study and characterization of this biomarker in pancreatic cancer. However, further research is needed to fully understand PIVKA-II’s implications and potential therapeutic targets.

**Table 1** summarizes the most relevant evidence on Molecular and Circulating biomarkers in PDAC.

**Table 1:** A comprehensive overview of the clinical utility and established roles of molecular and circulating biomarkers in PDAC.

Molecular Biomarkers			
	Marker	Employ	Role in PDAC
Key Molecular	KRAS (95%)	Diagnosis- Target Therapy	Proliferation, metabolic reprogramming, immune escape (11).
	TP53 (50-75%)	Diagnosis	Key mediator of TGF-β signaling, cancer development (22).
	SMAD4 (90%)	Diagnosis	Cell grow, differentiation and apoptosis (28).
	CDKN2A(95%)	Diagnosis	Proliferation and chemotherapy resistance (38).
New Potential	BRCA1/2	Diagnosis-Prognosis	DNA repair and tumor suppression (30).
	MLL	Target Therapy	Regulation of PDL-1 expression and immune evasion (47, 48).
	Gastrokine	Diagnosis-Prognosis	Desmoplastic-stromal changes, proliferation rate (50, 51).
	Nucleoporins	Diagnosis-TargetTherapy	Malignant transformation; different PDAC subtypes (55).
	CCNB1	Prognosis-Target Therapy	Microenvironment (40).
	FHL	Target Therapy	Survival, proliferation, and radioresistance (62).
	HLA-DPA1	Target Therapy	Loss of heterozygosity (LOH) immune escape (65).
	TUBB1	Target Therapy	Tumor progression (66).

Circulating Biomarkers			
	Marker	Employ	Role in PDAC
Recommended	CA19.9 *	Diagnosis-Target Therapy	Cancer progression by glycosylating proteins (70, 73).
	CEA	Diagnosis	Help to discriminate benign/ malignant pancreatic (79).
New Potential	MMPs	Diagnosis	ECM remodeling, tumor progression, cytokine regulation (80, 82).
	TIMP1	Diagnosis-Target Therapy	Formation of Neutrophil extracellular traps (NETs) (86).
	GDF15	Diagnosis	Tumor development and chemoresistance (90, 91).
	PIVKAII	Diagnosis Target Therapy and Follow-up	Decrease after surgical pancreatic removal; in vitro secretion correlates with glucose-induced EMT (107, 108).

*Gold Standard.

## 4 Conclusion

Pancreatic ductal adenocarcinoma (PDAC) remains a challenging malignancy with a poor prognosis, emphasizing the need for effective biomarkers to enhance diagnosis, treatment selection, and patient outcomes (46). Recent advances in molecular profiling have identified potential biomarkers for early diagnosis, prognosis, and targeted therapies (11, 111). Molecular markers such as KRAS and SMAD4, and novel circulating markers like GDF-15, PIVKA-II, and TIMP-1, show promise in enhancing diagnostic accuracy and prognostic evaluations. Additionally, genetic alterations in KRAS G12C, BRCA1/2, and HLA-DPA1 are emerging as predictive biomarkers for targeted treatments, including PARP inhibitors and immunotherapies (46, 112). As seen from the exploration of various emerging molecular and circulating biomarkers, research is increasingly focusing on proteins involved in the interplay between the tumor and its microenvironment. This shift underscores the importance of understanding the dynamic interactions within the tumor microenvironment, which are crucial in cancer progression and metastasis. As research advances, the integration of molecular and circulating biomarkers into clinical practice holds the potential to enable more personalized and effective treatment strategies for PDAC patients (46, 113, 114). In addition to the emerging biomarkers discussed, serum ctDNA levels have also shown great promise for diagnosing PDAC, detecting minimal residual disease (MRD), evaluating therapeutic response, and facilitating early detection of recurrence. ctDNA offers greater sensitivity and specificity compared to traditional antigen biomarkers, although standard clinical guidelines for its use are still under review and validation (9).

**Figure 1:**
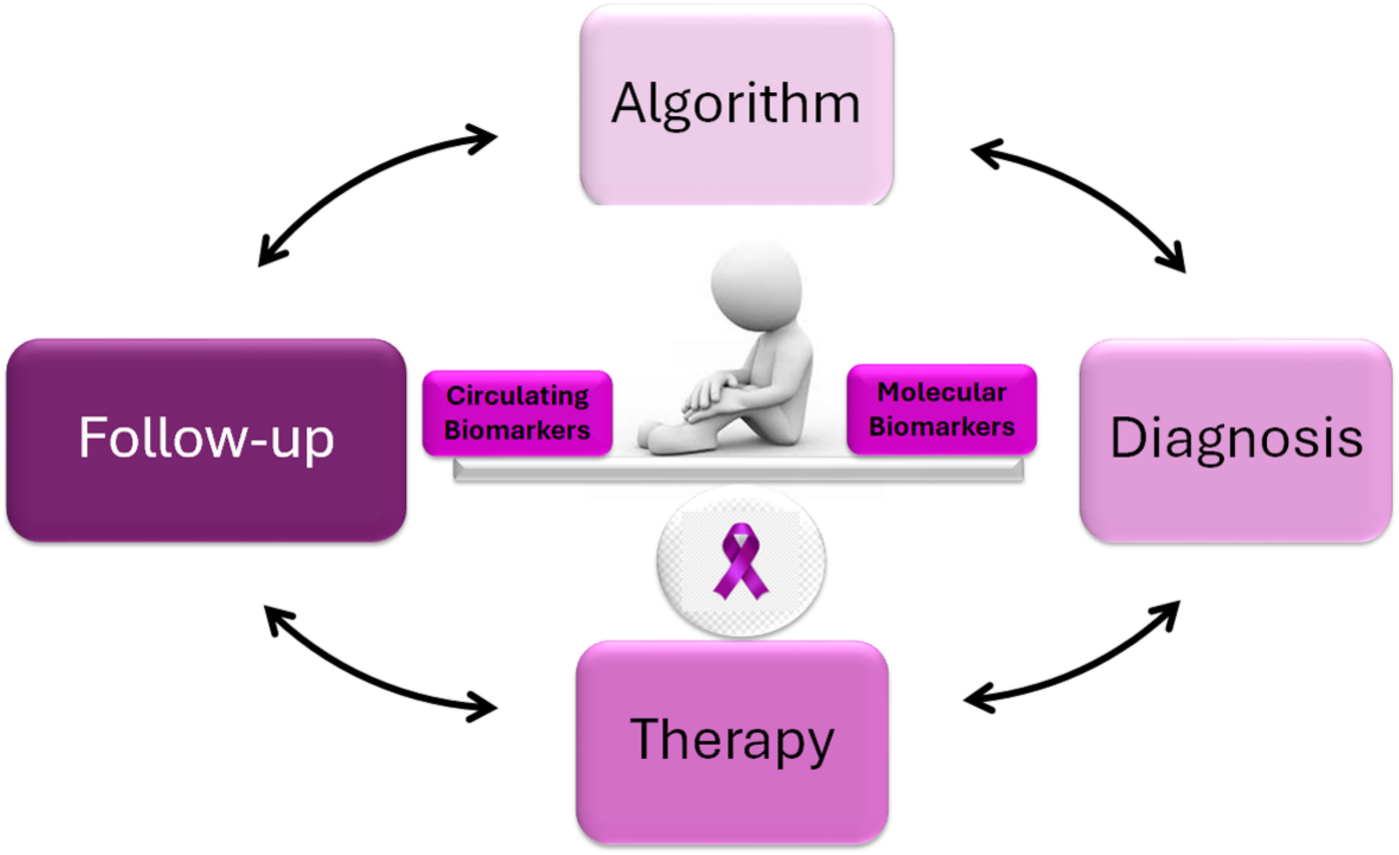
Features of PDAC Molecular and Circulating Biomarkers. The interplay between molecular and circulating biomarkers could represent a new strategy in the management of PDAC.

Finally, the development of sophisticated diagnostic algorithms incorporating these biomarkers could revolutionize PDAC management. Artificial intelligence (AI), combined with these algorithms, can analyze complex datasets by integrating molecular, genetic, and clinical information, resulting in more precise disease management. In conclusion, this mini-review underscores the importance of emerging molecular and circulating biomarkers, alongside the necessity of integrating them into diagnostic algorithms. Such integration aims to directly translate research findings into clinical practice, establishing a comprehensive approach to managing PDAC patients at all disease stages (**Figure 1**). Ongoing research and technological advancements will be essential to improving the early diagnosis and treatment of PDAC.
